# Bipolar and obsessive-compulsive disorders psychopathological intersection: An exploratory study

**DOI:** 10.1192/j.eurpsy.2023.535

**Published:** 2023-07-19

**Authors:** Y. A. Ferrao, M. Bertoluci, L. S. Boff, H. Beckhauser, I. Ghiorzi, G. Langa

**Affiliations:** Clinical Neurosciences, Porto Alegre Health Sciences Federal University (UCSPA), Porto Alegre, Brazil

## Abstract

**Introduction:**

Bipolar Mood Disorder (BD) and Obsessive-Compulsive Disorder (OCD) are psychiatric conditions that frequently co-occur and express a challenging phenomenology for treatment and diagnosis, since obsessive-compulsive symptoms tend to fluctuate according to mood phases of BD patients. Understanding the shared psychopathology of this comorbidity has relevant implications for the treatment of these patients, and the hypothesis that BD and OCD would have a shared neurobiology is currently being discussed. Most studies of this comorbidity have examined differences between BD and BD/OCD patients or between OCD and BD/OCD patients. This study aimed to analyze in detail the clinical, phenomenological and psychopathological characteristics of patients with BD, OCD, and BD/OCD.

**Objectives:**

This study aimed to analyze in detail the clinical, phenomenological andpsychopathological characteristics of patients with BD, OCD, and BD/OCD.

**Methods:**

This study consisted of a sample of 21 BD patients, 21 OCD patients and 21 BD/OCD patients who underwent the application of the MINI, Y-BOCS, DY-BOCS, HAM-D, HAM-A, YMRS, of Sensory Phenomena (USP), as well as questions about sociodemographic characteristics, personal and family psychiatric history. We performed the YBOCS scale asking patients with BD to respond 3 times the scale: in the current time (during euthimya) and retrospectively for previous manic or depressive episodes.

**Results:**

BD/OCD group had a higher rate of having stopped working due to comorbid disorders, a higher history of family suicide attempt and completed family suicide, a higher prevalence of substance use disorder in the family, and a higher prevalence of hoarding symptoms. In the BD sample, 47,6% had obsessive-compulsive symptoms. The presence of OCD conferred a higher prevalence of sensory phenomena. Patients reported a 19% (median, 0.19, range -1.00 to 1.88) worsening of OCD during depression, and a 9.5% worsening (median, 0.095, range of -1.00 to 1.36) during the manic phase.

**Conclusions:**

The results suggest that BD/OCD patients have greater loss of functionality, higher rates of hoarding symptoms, family history with greater suicidality and higher rates of substance use disorder (SUD) and worsening of OCD in both mania and depression. The psychopathological findings of this study allow us to conclude that BD/OCD patients have higher morbidity.

LIMITATIONS: Small size sample and possible recall bias in the interview, as questions wereasked retrospectively.

**Disclosure of Interest:**

None Declared

